# Incidence of ARDS and outcomes in hospitalized patients with COVID-19: a global literature survey

**DOI:** 10.1186/s13054-020-03240-7

**Published:** 2020-08-21

**Authors:** Susan J. Tzotzos, Bernhard Fischer, Hendrik Fischer, Markus Zeitlinger

**Affiliations:** 1 Apeptico GmbH, Mariahilfer Strasse 136, 1150 Vienna, Austria; 2 Department of Clinical Pharmacology, Medical University of Vienna, Waehringer Guertel 18-20, 1090 Vienna, Austria

**Keywords:** ARDS, COVID-19, Hospital, ICU, Incidence, Mortality, SARS-CoV-2

Coronavirus disease 2019 (COVID-19) caused by severe acute respiratory syndrome coronavirus 2 (SARS-CoV-2) appeared just over 7 months ago in Wuhan, China. Early reports from China indicated that although some cases are asymptomatic, 20% of COVID-19 cases follow a severe course, necessitating hospitalization, with a quarter of hospitalized patients requiring intensive care unit (ICU) facilities [1]. Later reports from China and other countries substantiated these data, although ICU admission rates, proportion of patients receiving invasive mechanical ventilation (IMV), and mortality rates differ considerably between studies [2].

The life-threatening form of respiratory failure, acute respiratory distress syndrome (ARDS) is a frequent complication in COVID-19 [3]. The severity of ARDS is classified into categories of mild, moderate, and severe, depending on the degree of hypoxemia [4]. Patients with moderate-to-severe ARDS require invasive mechanical ventilation (IMV) and have a poor prognosis [4]. The incidence of ARDS and specifically, moderate-to-severe ARDS, among COVID-19 patients is currently unknown [5].

We describe here the results of a survey of clinical studies reporting COVID-19-associated ARDS in hospitalized patients since the beginning of the COVID-19 pandemic in January until the end of July 2020. Our aim was to obtain a clearer picture of the incidence of COVID-19-associated ARDS in hospitalized patients on a global level, to better define the burden to healthcare systems and to inform critical care clinicians. This information should enable the prediction of requirements for hospital resources and thereby facilitate planning an appropriate and timely response in the future.

We carried out regular searches of PubMed using combinations of the search terms “ARDS,” “COVID-19,” “clinical characteristics,” “clinical features,” “clinical findings,” “ICU,” “incidence,” “outcome,” and “prevalence” (last search July 31, 2020).

Over 1000 publications were retrieved from which only studies reporting consecutively hospitalized patients, and giving numbers for ARDS patients and outcomes, were selected. Meta-analyses were excluded.

Seventeen studies reporting results from 2486 hospitalized COVID-19 patients in five countries fitted the inclusion criteria (Tables 1 and 2). Limitations are that seven studies did not define ARDS and only one study classified patients as mild, moderate, and/or severe; the patient sample is comparatively small: twelve of the studies had less than 200 patients. Furthermore, there was heterogeneity in types of data gathered by each research group, hence for many of the studies, patient numbers did not permit calculation of all parameters (Tables 1 and 2).

**Table 1:** Incidence of ARDS, ICU admission, IMV treatment, and outcome in hospitalized COVID-19 patients

Study author, online publication date, doi	Location	Number of patients, *N*	Patients with ARDS	Patients transferred to ICU	Patients who received IMV	Mortality hospitalized patients
Huang C, 24/1; doi: 10.1016/S0140-6736(20)30183-5	Wuhan, China	41	12/41 (29)	13/41 (32)	4/41 (10)	6/41 (15)
Wang D, 7/2; doi: 10.1001/jama.2020.1585	Wuhan, China	138	27/138 (19)	36/138 (26)	17/138 (12)	6/138 (4)
Xu X,19/2; doi: 10.1136/bmj.m606	Zhejiang, China	62	1/62 (2)	1/62 (2)	1/62 (2)	0/62 (0)
Zhou F, 11/3; doi: 10.1016/S0140-6736(20)30566-3	Wuhan, China	191	59/191 (31)	50/191 (26)	32/191 (17)	54/191 (28)
Wu C, 13/3; doi: 10.1001/jamainternmed.2020.0994	Wuhan, China	201	84/201 (42)	53/201 (26)	6/201 (3)	44/201 (22)
Dreher M, 17/4; doi: 10.3238/arztebl.2020.0271	Aachen, Germany	50	24/50 (48)	24/50 (48)	24/50 (48)	7/50 (14)
Yao Q, 24/4; doi: 10.20452/pamw.15312	Huanggang, China	108	45/108 (42)	17/108 (16)	10/108 (9)	12/108 (11)
Aggarwal S, 26/4; doi: 10.1515/dx-2020-0046	Des Moines, Iowa, USA	16	5/16 (31)	8/16 (50)	5/16 (31)	3/16 (19)
Inciardi R, 8/5; doi: 10.1093/eurheartj/ehaa388	Brescia, Italy	99	19/99 (19)	12/99 (12)	2/99 (2)	26/99 (26)
Hong K, 11/5; doi: 10.3349/ymj.2020.61.5.431	Daegu, South Korea	98	18/98 (18)	13/98 (13)	11/98 (11)	5/98 (5)
Yang L, 26/5; doi: 10.1016/j.jcv.2020.104475	Yichang, China	200	32/200 (16)	29/200 (15)	16/200 (8)	15/200 (7)
Suleyman G, 1/6; doi: 10.1001/jamanetworkopen.2020.12270	Detroit, Michigan, USA	355	111/355 (31)	141/355 (40)	114/355 (32)	72/355 (20)
Zheng Y, 10/4; doi: 10.1016/j.jcv.2020.104366	Chengdu, China	99	15/99 (15)	32/99 (32)	NA	3/99 (3)
Chen N, 30/1; doi: 10.1016/S0140-6736(20)30211-7	Wuhan, China	99	17/99 (17)	NA	NA	11/99 (11)
Chen T, 26/3; doi: 10.1136/bmj.m1091	Wuhan, China	274^a^	196(NA)^a^	NA	NA	113(NA)^a^
Yu T, 27/4; doi: 10.1016/j.clinthera.2020.04.009	Dongguan, China	95	24/95 (25)	NA	NA	1/95 (1)
Ciceri F, 12/6; doi: 10.1016/j.clim.2020.108509	Milan, Italy	360	245/360 (68)	NA	NA	82/360 (23)
Total (weighted average)		2486	738/2212 (33)^b^	429/1658 (26)	242/1559 (16)	347/2212 (16)^b^

Data are presented as *n*/*N*(%)

*Abbreviations*: *ARDS* acute respiratory distress syndrome, *COVID-19* coronavirus disease 2019, *ICU* intensive care unit, *IMV* invasive mechanical ventilation, *NA* not available: insufficient data for calculation

^a^From a hospitalized cohort of 799 COVID-19 patients, data available for 274 patients: 113 deaths and 161 recovered

^b^Patient numbers for Chen T study not included

**Table 2:** Incidence of ARDS, IMV treatment, and outcomes in COVID-19 patients admitted to an ICU

Study author, online publication date^a^	Location	ICU patients with ARDS	ICU patients who received IMV	Mortality ICU patients	Mortality ARDS patients	Mortality IMV patients	Prevalence of ARDS in non-survivors
Dreher M, 17/4	Aachen, Germany	24/24 (100)	24/24 (100)	3/24 (13)	3/24 (13)	3/24 (13)	3/7 (43)
Yao Q, 24/4	Huanggang, China	17/17 (100)	10/17 (59)	12/17 (71)	12/45 (27)	10/10 (100)	12/12 (100)
Hong K, 11/5	Daegu, South Korea	13/13 (100)	11/13 (85)	4/13 (31)	4/18 (22)	NA	4/5 (80)
Xu X,19/2	Zhejiang, China	1/1 (100)	1/1 (100)	0/1 (0)	0/1 (0)	0/1 (0)	NA
Wu C, 13/3	Wuhan, China	NA	6/53 (11)	NA	44/84 (52)	6/6 (100)	44/44 (100)
Zhou F, 11/3	Wuhan, China	NA	NA	39/50 (78)	50/59 (85)	31/32 (97)	50/54 (93)
Zheng Y, 10/4	Chengdu, China	15/32 (47)	NA	3/32 (9)	3/15 (20)	NA	3/3 (100)
Suleyman G, 1/6	Detroit, Michigan, USA	104/141 (74)	114/141 (81)	57/141 (40)	NA	52/114 (46)	NA
Huang C, 24/1	Wuhan, China	11/13 (85)	4/13 (31)	5/13 (38)	NA	NA	NA
Wang D, 7/2	Wuhan, China	22/36 (61)	21/36 (58)	6/36 (17)	NA	NA	NA
Yang L, 26/5	Yichang, China	21/29 (72)	16/29 (55)	14/29 (48)	NA	NA	NA
Chen T, 26/3	Wuhan, China	NA	NA	NA	113/196 (58)	17/17 (100)	113/113 (100)
Aggarwal S, 26/4	Des Moines, Iowa, USA	NA	5/8 (63)	3/8 (38)	NA	NA	NA
Inciardi R, 8/5	Brescia, Italy	NA	NA	NA	17/19 (89)	NA	17/26 (65)
Chen N, 30/1	Wuhan, China	NA	NA	NA	11/17 (65)	NA	11/11 (100)
Ciceri F, 12/6	Milan, Italy	NA	NA	NA	65/245 (27)	NA	65/82 (79)
Yu T, 27/4	Dongguan, China	NA	NA	NA	NA	NA	NA
Total (weighted average)		228/306 (75)	212/335 (63)	146/363 (40)	322/722 (45)	119/203 (59)	322/357 (90)

Data are presented as *n*/*N*(%)

*Abbreviations*: *ARDS* acute respiratory distress syndrome, *COVID-19* coronavirus disease 2019, *ICU* intensive care unit, *IMV* invasive mechanical ventilation, *NA* not available: insufficient data for calculation

^a^For study reference see Table 1

There is variability between individual studies with respect to frequency of ARDS, rates of ICU admission, and mortality among patients. Calculation of weighted averages for these parameters incorporating data from individual studies for which data is available indicate that among hospitalized COVID-19 patients, approximately 1/3 (33%) develop ARDS, 1/4 (26%) require transfer to an ICU, 1/6 (16%) receive IMV, and 1/6 (16%) die (Table 1). For COVID-19 patients transferred to an ICU, nearly 2/3 (63%) receive IMV and 3/4 (75%) have ARDS (Table 2). The mortality rate of ICU COVID-19 patients is 40% and of those who receive IMV 59%; the mortality rate in COVID-19-associated ARDS is 45%, and the incidence of ARDS among non-survivors of COVID-19 is 90% (Table 2). The high incidence of ARDS among COVID-19 patients revealed in our survey is consistent with the results of postmortem examinations of patients with COVID-19, in which the predominant finding is diffuse alveolar damage, the most frequent histopathologic correlate of ARDS.

For as long as there is neither a safe and efficacious vaccine nor therapy for severely affected COVID-19 patients, standard supportive care with lung-protective mechanical ventilation will be the cornerstone of treatment for these patients [5, 6]. The implications of these survey results are important and demonstrate the considerable challenges posed by the “COVID-19 crisis” to ICU practitioners, hospital administrators, and health policy makers.

Susan Tzotzos, MSc, PhD

Bernhard Fischer, PhD

Hendrik Fischer, PhD

Markus Zeitlinger, MD, PhD

## Data Availability

Data generated or analyzed during this study are included in this published article.

## Abbreviations

ARDS: Acute respiratory distress syndrome
COVID-19: Coronavirus disease 2019
ICU: Intensive care unit
IMV: Invasive mechanical ventilation
SARS-CoV-2: Coronavirus 2

## References

[1] Wu Z, McGoogan JM. Characteristics of and important lessons from the coronavirus disease 2019 (COVID-19) outbreak in China: summary of a report of 72314 cases from the Chinese Center for Disease Control and Prevention. JAMA. 2020. 10.1001/jama.2020.2648. 10.1001/jama.2020.2648 32091533

[2] Wunsch H (2020). Mechanical ventilation in COVID-19: interpreting the current epidemiology. Am J Respir Crit Care Med.

[3] Wu C, Chen X, Cai Y, et al. Risk factors associated with acute respiratory distress syndrome and death in patients with coronavirus disease 2019 pneumonia in Wuhan, China. JAMA Intern Med. 2020. 10.1001/jamainternmed.2020.0994. 10.1001/jamainternmed.2020.0994 PMC7070509 32167524

[4] Definition Task Force ARDS, Ranieri VM, Rubenfeld GD (2012). Acute respiratory distress syndrome: the Berlin Definition. JAMA..

[5] Fan E, Beitler JR, Brochard L, et al. COVID-19-associated acute respiratory distress syndrome: is a different approach to management warranted? Lancet Respir Med. 2020:S2213–2600(20)30304–0. 10.1016/S2213-2600(20)30304-0. 10.1016/S2213-2600(20)30304-0 PMC7338016 32645311

[6] Matthay MA, Aldrich JM, Gotts JE (2020). Treatment for severe acute respiratory distress syndrome from COVID-19. Lancet Respir Med.

